# New Coleoptera records from New Brunswick, Canada: Lycidae

**DOI:** 10.3897/zookeys.179.2494

**Published:** 2012-04-04

**Authors:** Reginald P. Webster, Jon D. Sweeney, Ian DeMerchant

**Affiliations:** 1 Natural Resources Canada, Canadian Forest Service - Atlantic Forestry Centre, 1350 Regent St., P.O. Box 4000, Fredericton, NB, Canada E3B 5P7

**Keywords:** Lycidae, new records, Canada, New Brunswick

## Abstract

Eight species of Lycidae are newly recorded from New Brunswick, Canada, bringing the total number of species known from the province to 16. The first documented records from New Brunswick are provided for *Greenarius thoracicus* (Randall) *Erotides scuptilis* (Say), and *Calopteron terminale* (Say) reported by Majka et al. (2011). *Eropterus arculus* Green, *Lopheros crenatus* (Germar), and *Calochromus perfacetus* (Say) are reported for the first time in the Maritime provinces. Collection data, habitat data, and distribution maps are presented for all these species.

## Introduction

The Lycidae of North America was reviewed by Green 1949, 1950, 1951, 1952, 1953, 1954), and all species in the Maritime provinces of Canada can readily be determined using these keys. Larvae of the Lycidae are usually found in rotten logs, in leaf litter, and under bark and probably feed on myxomycetes or metabolic products of fungi (Lawrence 1982; Miller 2002). Adults are usually found on leaves or flowers and feed on nectar and honeydew. Larvae and adults are distasteful, and adults are often brightly colored and probably aposomatic in coloration (Miller 2002).

Twenty-nine species are known from Canada and five were reported from New Brunswick by McNamara (1991). Majka et al. (2011) reported *Greenarius thoracicus* (Randall) *Erotides scuptilis* (Say), and *Calopteron terminale* (Say) as occurring in New Brunswick but did not provide any supporting references or data. Here, eight species of Lycidae are newly reported from New Brunswick, Canada, as well as the first documented records for *Greenarius thoracicus* (Randall) *Erotides scuptilis* (Say), and *Calopteron terminale* (Say).

## Methods and conventions

The following records are based in part on specimens collected as part of a general survey by the first author to document the Coleoptera fauna of New Brunswick.

## Collection methods

Various collection methods were employed to collect the Lycidae reported in this study. Details are outlined Webster et al. (2009, Appendix). A number of specimens were also collected as by-catch in Lindgren 12-funnel traps (ConTech Inc., Delta, BC) baited with various attractants as part of a study to develop a general attractant for the detection of invasive species of Cerambycidae. These traps mimic tree trunks and are often effective for sampling species of Coleoptera that live in microhabitats associated with standing trees (Lindgren 1983). Details on the methods used for deployment of these traps are outlined in Webster et al. (in press). A description of the habitat was recorded for all specimens collected during this survey. Locality and habitat data are presented exactly as on labels for each record. This information, as well as additional collecting notes, is summarized in collection and habitat data for each species.

## Specimen preparation

Males of some species of Lycidae (all *Plateros* sp.) were dissected to confirm their identity. The genital structures were dehydrated in absolute alcohol and mounted on points and then pinned with the specimens from which they originated.

## Distribution

Distribution maps, created using ArcMap and ArcGIS, are presented for each species in New Brunswick. Every species is cited with current distribution in Canada and Alaska, using abbreviations for the state, provinces, and territories. New records for New Brunswick are indicated in bold under Distribution in Canada and Alaska. The following abbreviations are used in the text:

Acronyms of collections examined or where specimens reside referred to in this study are as follows:

AFC Atlantic Forestry Centre, Natural Resources Canada, Canadian Forest Service, Fredericton, New Brunswick, Canada

CNC Canadian National Collection of Insects, Arachnids and Nematodes, Agriculture and Agri-Food Canada, Ottawa, Ontario, Canada

NBM New Brunswick Museum, Saint John, New Brunswick, Canada

RWC Reginald P. Webster Collection, Charters Settlement, New Brunswick, Canada

**Table:** 

**AK**	Alaska	**MB**	Manitoba
**YT**	Yukon Territory	**ON**	Ontario
**NT**	Northwest Territories	**QC**	Quebec
**NU**	Nunavut	**NB**	New Brunswick
**BC**	British Columbia	**PE**	Prince Edward Island
**AB**	Alberta	**NS**	Nova Scotia
**SK**	Saskatchewan	**NF & LB**	Newfoundland and Labrador*

## Results

Eight species of Lycidae are newly recorded from New Brunswick, Canada, bringing the total number of species known from the province to 16 (Table 1). The first documented records from New Brunswick are provided for *Greenarius thoracicus* (Randall) *Erotides scuptilis* (Say), and *Calopteron terminale* (Say) reported by Majka et al. (2011). *Eropterus arculus* Green, *Lopheros crenatus* (Germar), and *Calochromus perfacetus* (Say) are reported for the first time in the Maritime provinces (New Brunswick, Nova Scotia, Prince Edward Island).

**Table 1.** Species of Lycidae recorded from New Brunswick.

Family Lycidae Laporte
Subfamily Dictyopterinae Houlbert
Tribe Dictyopterini Houlbert
*Greenarius thoracicus* (Randall)
*Dictyopterus aurora* (Herbst)
Subfamily Lycinae Laporte
Tribe Erotini LeConte
*Eropterus arculus* Green**
*Eros humeralis* (Fabricius)*
*Lopheros crenatus* (Germar)**
*Lopheros fraternus* (Randall)*
*Erotides sculptilis* (Say)
Tribe Calochromini Lacordaire
*Calochromus perfacetus* (Say)**
Tribe Calopterini Green
*Caenia dimidiata* (Fabricius)
*Calopteron terminale* (Say)
*Leptoceletes basali*s LeConte
Tribe Platerodini Kleine
*Plateros bispiculatus* Green*
*Plateros flavoscutellatus* Blatchley*
*Plateros lictor* (Newman)
*Plateros subfurcatus* Green*
*Plateros volatus* Green

**Notes:** *New to province; **New to Maritime provinces

### Species accounts

All records below are species newly recorded from New Brunswick, Canada unless noted otherwise (additional records). Species followed by ** are newly recorded from the Maritime provinces.

The classification of the Lycidae follows Kazantsev (2004) and Bouchard et al. (2011).

#### Family Lycidae, Laporte, 1836. Subfamily Dictyopterinae Houlbert, 1922. Tribe Dictyopterini Houlbert, 1922

##### Greenarius thoracicus

(Randall, 1838)

http://species-id.net/wiki/Greenarius_thoracicus

Map 1

###### Material examined.

**Additional New Brunswick records. Charlotte Co.**, 10 km NW of New River Beach, 45.2110°N, 66.6170°W, 29.VI–16.VII.2010, R. Webster & C. MacKay, old growth eastern white cedar forest, Lindgren funnel traps (2, RWC). **Queens Co.**, Cranberry Lake P.N.A. (Protected Natural Area), 46.1125°N, 65.6075°W, 10–15.VII.2009, R. Webster & M.-A. Giguère, old red oak forest, Lindgren funnel trap (1, AFC); same locality data and forest type, 7–13.VII.2011, M. Roy & V. Webster, Lindgren funnel trap (1, RWC). **Sunbury Co.**, Acadia Research Forest, 45.9866°N, 66.3841°W, 24–30.VI.2009, R. Webster & M.-A. Giguère, red spruce forest with red maple and balsam fir, Lindgren funnel trap (1, RWC). **York Co.**, 15 km W of Tracy off Rt. 645, 45.6848°N, 66.8821°W, 29.VII-4.VIII.2009, R. Webster & M.-A. Giguère, old red pine forest, Lindgren funnel trap (1, RWC); 14 km WSW of Tracy, S of Rt. 645, 45.6741°N, 66.8661°W, 16–30.VI.2010, R. Webster & C. MacKay, old mixed forest with red and white spruce, red and white pine, balsam fir, eastern white cedar, red maple, and *Populus* sp., Lindgren funnel trap (1, RWC).

###### Collection and habitat data.

Adults were captured in Lindgren funnel traps deployed in an old-growth eastern white cedar (*Thuja occidentalis* L.) forest, old red oak (*Quercus rubra* L.) forest, red spruce (*Picea rubens* Sarg.) forest, old red pine (*Pinus resinosa* Ait.) forest, and an old mixed forest. Adults were captured during June and July.

###### Distribution in Canada and Alaska.

BC, AB, MB, ON, QC, NB (Green 1951; McNamara 1991; Majka et al. 2011). *Greenarius thoracicus* was listed as occurring in New Brunswick by Majka et al. (2011) without any supporting references or data. Here we provide the first documented records from New Brunswick.

**Map 1.**
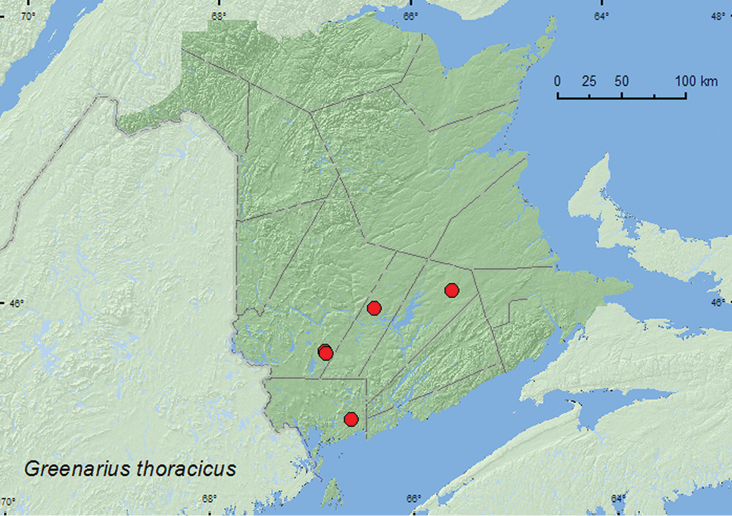
Collection localities in New Brunswick, Canada of *Greenarius thoracicus*.

#### Subfamily Lycinae Laporte, 1836. Tribe Erotini LeConte, 1881

##### Eropterus arculus

Green, 1951**

http://species-id.net/wiki/Eropterus_arculus

Map 2

###### Material examined.

**New Brunswick, Carleton Co.**, Jackson Falls, Bell Forest, 46.2200°N, 67.7231°W, 2.VIII.2004, R. P. Webster & M.-A. Giguère, mature hardwood forest, on foliage (1♂, NBM);Meduxnekeag Valley Nature Preserve, 46.1888°N, 67.6762°W, 4.VII.2005, R. P. Webster, river margin sweeping (1, RWC). **Queens Co.**, Cranberry Lake P.N.A, 46.1125°N, 65.6075°W, 29.VII-6.VIII.2009, R. Webster & M.-A. Giguère, old red oak forest, Lindgren funnel trap (1, RWC). **York Co.**,Charters Settlement, 45.8300°N, 66.7347°W, 29.VII.2004, R. P. Webster, regenerating mixed forest, on foliage (1, RWC); same locality but 45.8430°N, 66.7275°W, 12.VII.2005, 20.VII.2008, R. P. Webster, regenerating mixed forest, beating foliage (2, RWC).

###### Collection and habitat data.

*Eropterus arculus* adults were collected by beating or sweeping foliage and hand picking adults from foliage in mature hardwood forests with American beech (*Fagus grandifolia* Ehrh.) and sugar maple (*Acer saccharum* Marsh.), regenerating mixed forests, and along a river margin. One individual was captured in a Lindgren funnel trap deployed in an old red oak forest. Adults were collected during July and August.

###### Distribution in Canada and Alaska.

ON, QC, **NB** (McNamara 1991).

**Map 2.**
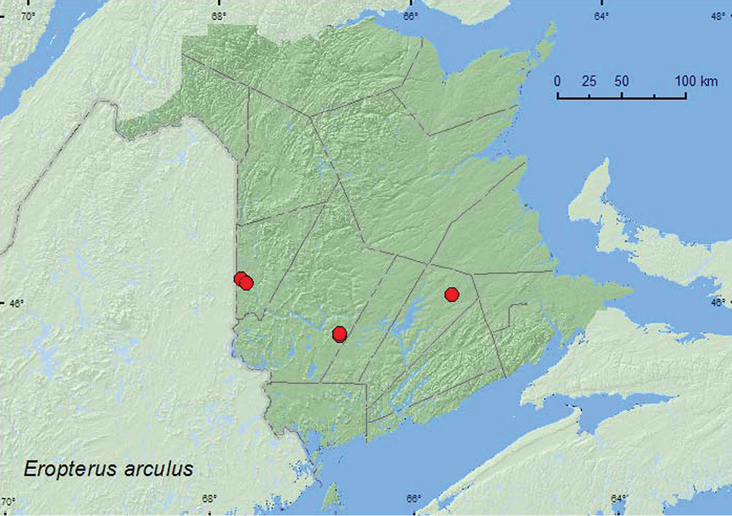
Collection localities in New Brunswick, Canada of *Eropterus arculus*.

##### Eros humeralis

(Fabricius, 1801)

http://species-id.net/wiki/Eros_humeralis

Map 3

###### Material examined.

**New Brunswick, Albert Co.**, Caledonia Gorge P.N.A., 45.8175°N, 64.7770°W, 6.VII.2011, R. P. Webster, mature hardwood forest, under bark of rotten sugar maple log (1, RWC). **Carleton Co.**, Jackson Falls, Bell Forest, 46.2200°N, 67.7231°W, 7–14.VII.2009, R. P. Webster & M.-A. Giguère, mature hardwood forest, Lindgren funnel trap (1, RWC). **Queens Co.**, Cranberry Lake P.N.A, 46.1125°N, 65.6075°W, 29.VI–7.VII.2011, M. Roy & V. Webster, old red oak forest, Lindgren funnel trap (1, RWC). **York Co.**, 15 km W of Tracy off Rt. 645, 45.6848°N, 66.8821°W, 29.VII-4.VIII.2009, R. Webster & M.-A. Giguère, old red pine forest, Lindgren funnel trap (1, RWC).

###### Collection and habitat data.

This species was captured in Lindgren funnel traps deployed in a mature hardwood forest (beech and sugar maple), an old red oak forest, and an old red pine forest. One adult was found under bark of a rotten sugar maple log in a mature hardwood forest (mostly sugar maple). Adults were captured during July and August.

###### Distribution in Canada and Alaska.

ON, QC, **NB**, NS (Green 1951; McNamara 1991; Dollin et al. 2008; Bishop et al. 2009).

**Map 3.**
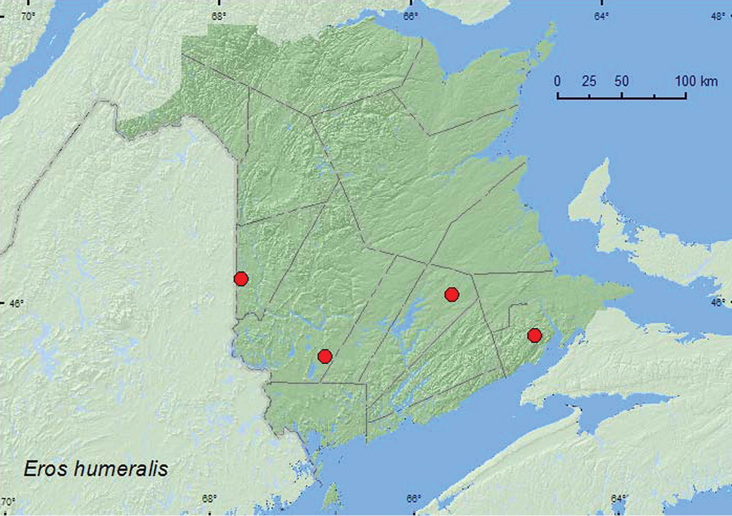
Collection localities in New Brunswick, Canada of *Eros humeralis*.

##### Lopheros crenatus

(Germar, 1824)**

http://species-id.net/wiki/Lopheros_crenatus

Map 4

###### Material examined

**. New Brunswick, Carleton Co.**, Meduxnekeag Valley Nature Preserve, 46.1940°N, 67.6800°W, 3.VII.2006, R. P. Webster, mixed forest, beating foliage (1, RWC).

###### Collection and habitat data.

One individual of *Lopheros crenatus* was collected from foliage during early July in a mixed forest.

###### Distribution in Canada and Alaska.

MB, ON, QC, **NB** (Green 1951; McNamara 1991).

**Map 4.**
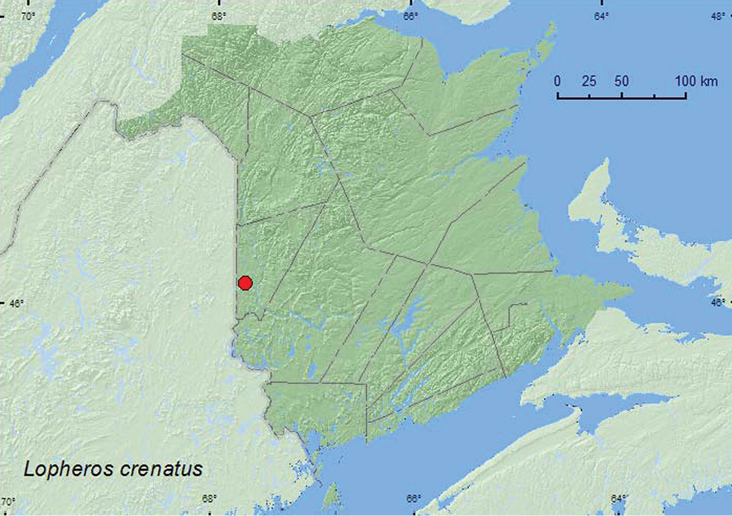
Collection localities in New Brunswick, Canada of *Lopheros crenatus*.

##### Lopheros fraternus

(Randall, 1838)

http://species-id.net/wiki/Lopheros_fraternus

Map 5

###### Material examined.

**New Brunswick, Queens Co.**, Cranberry Lake P.N.A, 46.1125°N, 65.6075°W, 10–15.VII.2009, R. Webster & M.-A. Giguère, old red oak forest, Lindgren funnel trap (1, RWC). **Sunbury Co.**, Acadia Research Forest, 45.9866°N, 66.3841°W, 13–21.VII.2009, R. Webster & M.-A. Giguère, red spruce forest with red maple and balsam fir, Lindgren funnel trap (1, RWC). **York Co.**, 15 km W of Tracy off Rt. 645, 45.6848°N, 66.8821°W, 30.VI–13.VII.2010, R. Webster & K. Burgess, old red pine forest, Lindgren funnel trap (1, RWC).

###### Collection and habitat data.

Adults werecaptured in Lindgren funnel traps deployed in a red spruce forest, old red oak forest, and old red pine forest. This species was collected during July in New Brunswick.

###### Distribution in Canada and Alaska.

ON, QC, **NB**, NS (McNamara 1991; Dollin et al. 2008).

**Map 5.**
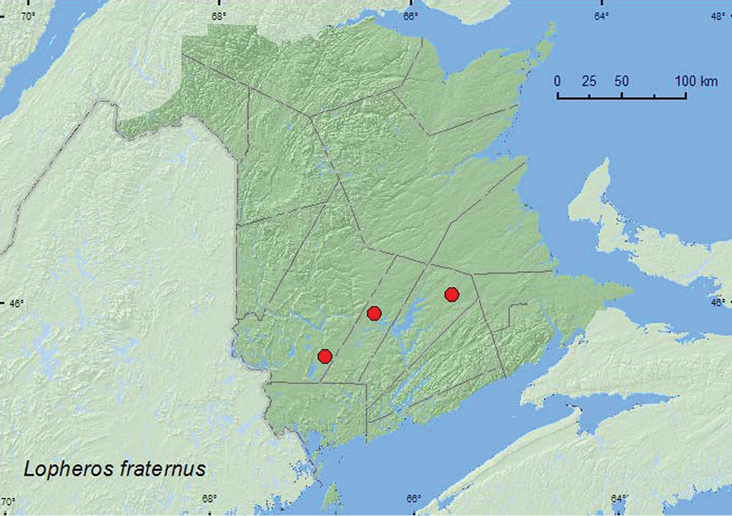
Collection localities in New Brunswick, Canada of *Lopheros fraternus*.

##### Erotides sculptilis

(Say, 1835)

http://species-id.net/wiki/Erotides_sculptilis

Map 6

###### Material examined.

**Additional New Brunswick records. Carleton Co.**, Meduxnekeag Valley Nature Preserve, 46.1957°N, 67.6803°W, 2.VIII.2004, R. P. Webster, mixed forest, beating foliage (1, RWC). **York Co.**,Charters Settlement, 45.8300°N, 66.7347°W, 29.VII.2004, R. P. Webster, regenerating mixed forest, on foliage (1, RWC); Charters Settlement, 45.8430°N, 66.7275°W, 17.VII.2007, R. P. Webster, regenerating mixed forest, sweeping foliage in brushy opening (1, RWC).

###### Collection and habitat data.

This species was collected by beating or sweeping foliage and hand picking adults from foliage in mixed and regenerating mixed forests during July and August.

###### Distribution in Canada and Alaska.

ON, QC, NB (Green 1951; McNamara 1991; Majka et al. 2011). *Erotides sculptilis* was listed as occurring in New Brunswick by Majka et al. (2011) without any supporting references or data. Here we provide the first documented records from New Brunswick.

**Map 6.**
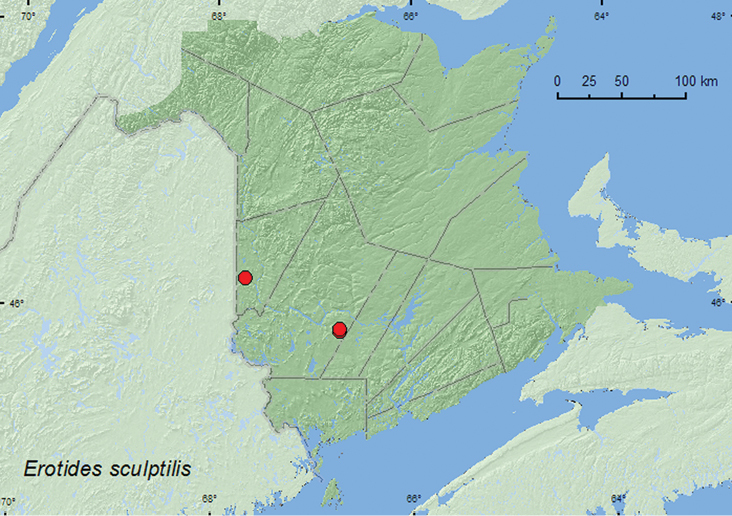
Collection localities in New Brunswick, Canada of *Erotides sculptilis*.

#### Tribe Calochromini Lacordaire, 1857

##### Calochromus perfacetus

(Say, 1825)**

http://species-id.net/wiki/Calochromus_perfacetus

Map 7

###### Material examined.

**New Brunswick, Carleton Co.**, Meduxnekeag Valley Nature Preserve, 46.1925°N, 67.6725°W, 13.VII.2004, R. P. Webster, mixed forest, beating foliage (1, RWC). **York Co.**,Charters Settlement, 45.8340°N, 66.7450°W, 25.VII.2006, R. P. Webster, mixed forest, on flowers of *Spiraea alba* (1, RWC).

###### Collection and habitat data.

Both individuals of *Calochromus perfacetus* from New Brunswick were collected in mixed forests, one from beating foliage, the other from flowers of *Spiraea alba* Du Roi. Adults were collected during July.

###### Distribution in Canada and Alaska.

SK, ON, QC, **NB** (McNamara 1991).

**Map 7.**
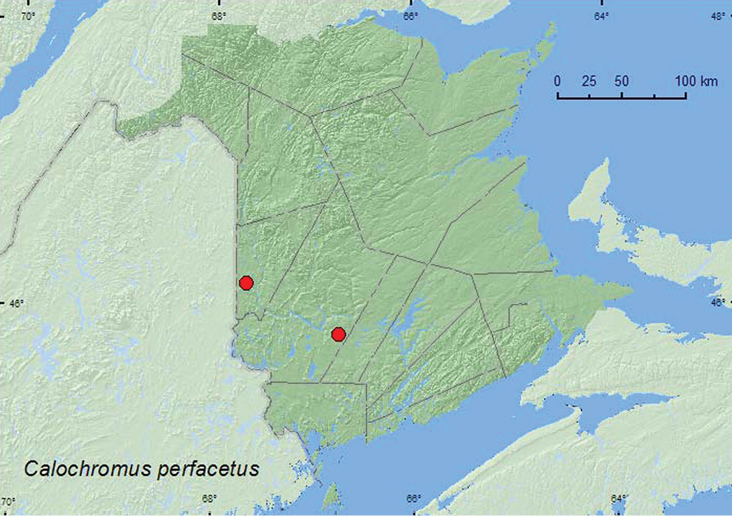
Collection localities in New Brunswick, Canada of *Calochromus perfacetus*.

#### Tribe Calopterini Green, 1949

##### Calopteron terminale

(Say, 1823)

http://species-id.net/wiki/Calopteron_terminale

Map 8

###### Material examined.

**Additional New Brunswick records. Queens Co.**, Waterborough, at boat landing at Grand Lake, 45.9072°N, 66.0127°W, 1.IX.2004, R. P. Webster, lakeshore in drift material (5, RWC); Cranberry Lake P.N.A, 46.1125°N, 65.6075°W, 14.VIII.2009, R. Webster & M.-A. Giguère, old red oak forest, on flowers of *Spiraea alba* (1, AFC); same locality data and forest type, 31.VIII-15.IX.2011, C. Hughs & R. Webster, Lindgren funnel trap (1, NBM). **Sunbury Co.**, Maugerville, Portobello Creek N.W.A., 45.8992°N, 66.4248°W, 28.VIII.2004, R. P. Webster, silver maple forest, on foliage (1, RWC). **York Co.**,Charters Settlement, 45.8430°N, 66.7275°W, 23.VIII.2003, 28.VIII.2004, 13.VIII.2004, R. P. Webster, regenerating mixed forest, on foliage (4, RWC); same locality but 45.8188°N, 66.7460°W, 11.IX.2004, R. P. Webster, clear-cut, under bark of conifer stump (2, RWC); Tracy, off Webb Rd., 45.6931°N, 66.6539°W, 31.VIII.2008, R. P. Webster, mixed forest, sweeping roadside vegetation (1, NBM).

###### Collection and habitat data.

*Calopteron terminale* (Say) was collected from drift material along a lakeshore, from (hand picking adults on) foliage in a silver maple (*Acer saccharinum* L.) forest, an old red oak forest, and regenerating mixed and mixed forests, and from under bark of a conifer stump in a clearcut. On individual was collected from flowers of *Spiraea alba*, another was captured in a Lindgren funnel trap. Adults were captured during August and September.

###### Distribution in Canada and Alaska.

MB, ON, QC, NB (Green 1952; McNamara 1991; Majka et al. 2011). *Calopteron terminale* was listed as occurring in New Brunswick by Majka et al. (2011) without any supporting references or data. Here we provide the first documented records from New Brunswick.

**Map 8.**
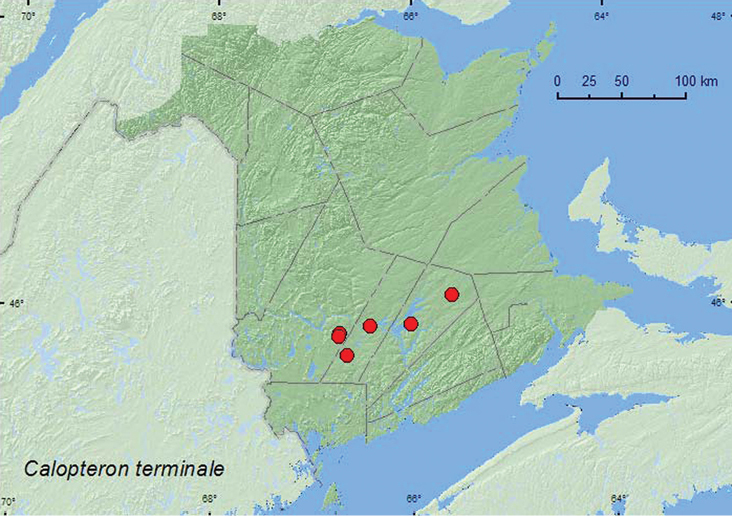
Collection localities in New Brunswick, Canada of *Calopteron terminale*.

#### Subfamily Platerodini Kleine, 1929

##### Plateros bispiculatus

Green, 1953

http://species-id.net/wiki/Plateros_bispiculatus

Map 9

###### Material examined.

**New Brunswick, York Co.**,Charters Settlement, 45.8430°N, 66.7275°W, 17.VII.2007, R. P. Webster, regenerating mixed forest, sweeping foliage in brushy opening (1, RWC).

###### Collection and habitat data.

One individual of this species was captured by sweeping foliage in a brushy opening in a regenerating mixed forest during July.

###### Distribution in Canada and Alaska.

ON, QC, **NB**, NS (Green 1953; McNamara 1991; Dollin et al. 2008).

**Map 9.**
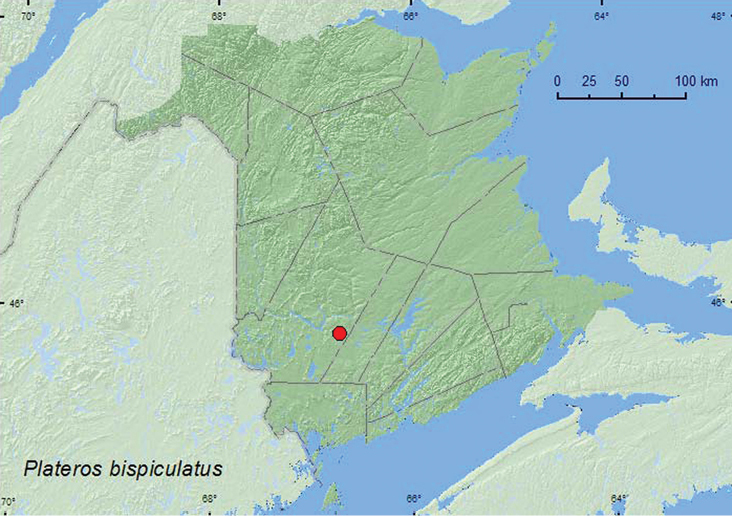
Collection localities in New Brunswick, Canada of *Plateros bispiculatus*.

##### Plateros flavoscutellatus

Blatchley, 1914

http://species-id.net/wiki/Plateros_flavoscutellatus

Map 10

###### Material examined.

**New Brunswick, Queens Co.**, Cranberry Lake P.N.A, 46.1125°N, 65.6075°W, 20.VII-4.VIII.2011, M. Roy & V. Webster, old red oak forest, Lindgren funnel trap (1, RWC). **York Co.**,Charters Settlement, 45.8395°N, 66.7391°W, 1.VIII.2004, R. P. Webster, mixed forest, u.v. light (1, RWC); same locality but 45.8430°N, 66.7275°W, 3.VIII.2004, 13.VIII.2004, R. P. Webster, regenerating mixed forest, sweeping foliage in brushy opening (2, RWC); 15 km W of Tracy off Rt. 645, 45.6848°N, 66.8821°W, 13–27.VII.2010, R. Webster & C. MacKay, old red pine forest, Lindgren funnel trap (1, RWC)

###### Collection and habitat data.

This species was captured in mixed and regenerating mixed forests, in an old red oak forest, and in an old red pine forest. Adults were collected at a black-light trap, by sweeping foliage, and in Lindgren funnel traps. Adults were collected during July and August.

###### Distribution in Canada and Alaska.

ON, QC, **NB**, NS (Green 1953; McNamara 1991).

**Map 10.**
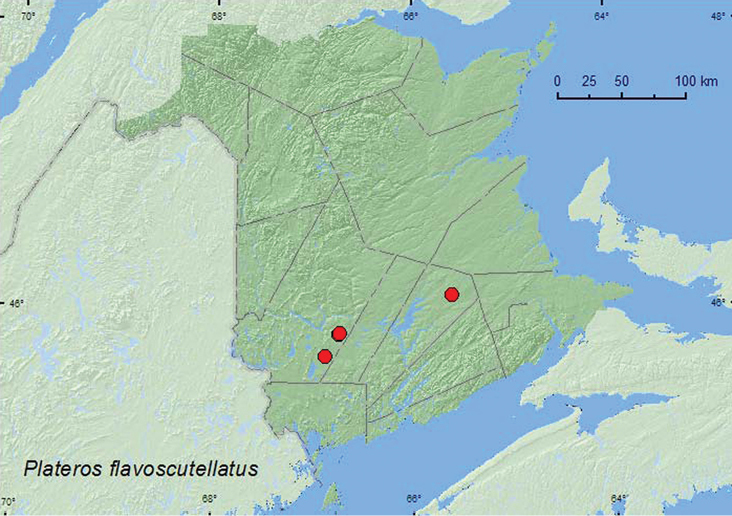
Collection localities in New Brunswick, Canada of *Plateros flavoscutellatus*.

##### Plateros subfurcatus

Green, 1953

http://species-id.net/wiki/Plateros_subfurcatus

Map 11

###### Material examined.

**New Brunswick, Sunbury Co.**, Acadia Research Forest, 45.9866°N, 66.3841°W, 30.VI–8.VII.2009, R. Webster & M.-A. Giguère, red spruce forest with red maple and balsam fir, Lindgren funnel trap (1, RWC). **York Co.**, Rt. 645 at Beaver Brook, 45.6830°N, 66.8679°W, 8.VII.2008, R. P. Webster, red maple and alder swamp, sweeping foliage (1, RWC).

###### Collection and habitat data.

One individual was captured in a Lindgren funnel trap deployed in a red spruce forest and another was collected by sweeping foliage in a red maple (*Acer rubrum* L.) and alder (*Alnus* sp.) swamp. Both adults were captured during July.

###### Distribution in Canada and Alaska.

ON, **NB**, NS (McNamara 1991; Bishop et al. 2009).

**Map 11.**
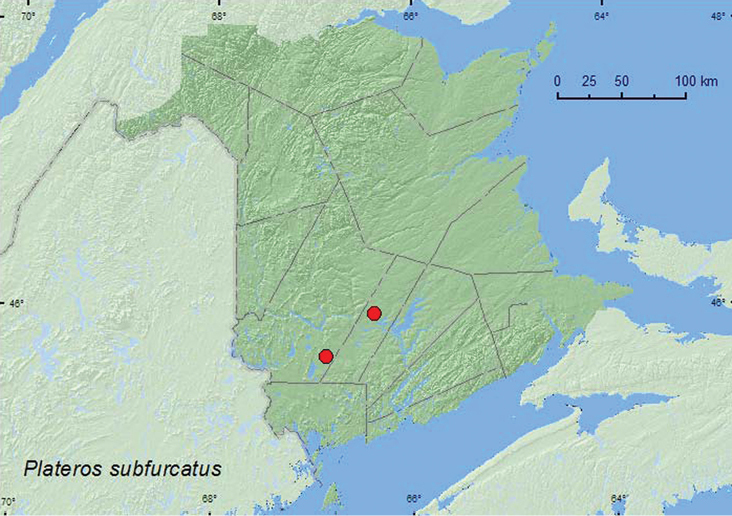
Collection localities in New Brunswick, Canada of *Plateros subfurcatus*.

## Supplementary Material

XML Treatment for Greenarius thoracicus

XML Treatment for Eropterus arculus

XML Treatment for Eros humeralis

XML Treatment for Lopheros crenatus

XML Treatment for Lopheros fraternus

XML Treatment for Erotides sculptilis

XML Treatment for Calochromus perfacetus

XML Treatment for Calopteron terminale

XML Treatment for Plateros bispiculatus

XML Treatment for Plateros flavoscutellatus

XML Treatment for Plateros subfurcatus
